# Comparison of accuracy of fibrosis degree classifications by liver biopsy and non-invasive tests in chronic hepatitis C

**DOI:** 10.1186/1471-230X-11-132

**Published:** 2011-11-30

**Authors:** Jérôme Boursier, Sandrine Bertrais, Frédéric Oberti, Yves Gallois, Isabelle Fouchard-Hubert, Marie-Christine Rousselet, Jean-Pierre Zarski, Paul Calès

**Affiliations:** 1 Liver-Gastroenterology department, University Hospital, Angers, France; 2 HIFIH laboratory, UPRES 3859, IFR 132, University, PRES UNAM, Angers, France; 3 Laboratory of Biochemistry and Molecular Biology, University Hospital, Angers, France; 4 Department of Cell and Tissue Pathology, University Hospital, Angers, France; 5 Liver-Gastroenterology department, University Hospital; INSERM/UJF U823, IAPC, IAB, University, Grenoble, France

## Abstract

**Background:**

Non-invasive tests have been constructed and evaluated mainly for binary diagnoses such as significant fibrosis. Recently, detailed fibrosis classifications for several non-invasive tests have been developed, but their accuracy has not been thoroughly evaluated in comparison to liver biopsy, especially in clinical practice and for Fibroscan. Therefore, the main aim of the present study was to evaluate the accuracy of detailed fibrosis classifications available for non-invasive tests and liver biopsy. The secondary aim was to validate these accuracies in independent populations.

**Methods:**

Four HCV populations provided 2,068 patients with liver biopsy, four different pathologist skill-levels and non-invasive tests. Results were expressed as percentages of correctly classified patients.

**Results:**

In population #1 including 205 patients and comparing liver biopsy (reference: consensus reading by two experts) and blood tests, Metavir fibrosis (F_M_) stage accuracy was 64.4% in local pathologists vs. 82.2% (p < 10^-3^) in single expert pathologist. Significant discrepancy (≥ 2F_M _vs reference histological result) rates were: Fibrotest: 17.2%, FibroMeter^2G^: 5.6%, local pathologists: 4.9%, FibroMeter^3G^: 0.5%, expert pathologist: 0% (p < 10^-3^). In population #2 including 1,056 patients and comparing blood tests, the discrepancy scores, taking into account the error magnitude, of detailed fibrosis classification were significantly different between FibroMeter^2G ^(0.30 ± 0.55) and FibroMeter^3G ^(0.14 ± 0.37, p < 10^-3^) or Fibrotest (0.84 ± 0.80, p < 10^-3^). In population #3 (and #4) including 458 (359) patients and comparing blood tests and Fibroscan, accuracies of detailed fibrosis classification were, respectively: Fibrotest: 42.5% (33.5%), Fibroscan: 64.9% (50.7%), FibroMeter^2G^: 68.7% (68.2%), FibroMeter^3G^: 77.1% (83.4%), p < 10^-3 ^(p < 10^-3^). Significant discrepancy (≥ 2 F_M_) rates were, respectively: Fibrotest: 21.3% (22.2%), Fibroscan: 12.9% (12.3%), FibroMeter^2G^: 5.7% (6.0%), FibroMeter^3G^: 0.9% (0.9%), p < 10^-3 ^(p < 10^-3^).

**Conclusions:**

The accuracy in detailed fibrosis classification of the best-performing blood test outperforms liver biopsy read by a local pathologist, i.e., in clinical practice; however, the classification precision is apparently lesser. This detailed classification accuracy is much lower than that of significant fibrosis with Fibroscan and even Fibrotest but higher with FibroMeter^3G^. FibroMeter classification accuracy was significantly higher than those of other non-invasive tests. Finally, for hepatitis C evaluation in clinical practice, fibrosis degree can be evaluated using an accurate blood test.

## Background

Whatever the diagnostic means, liver fibrosis is usually described in a synthetic, ordered manner, e.g., fibrosis classification. The development of histological classifications, i.e., Metavir fibrosis (F_M_) [1] or Ishak [2] semi-quantitative staging systems, was an initial step in this field. These histological classifications permitted the development of several non-invasive tests for the diagnosis of liver fibrosis, mainly due to hepatitis C virus (HCV). For statistical reasons, these tests were constructed for binary diagnoses such as significant fibrosis (i.e., bridging fibrosis) and included two classes of fibrosis stages (for example, F_M_0/1 vs. F_M_2/3/4). However, these broad classifications are less precise than the original histological classification. The prognostic interest of detailed fibrosis classification has been demonstrated [3]. Therefore, more detailed classifications reflecting histological fibrosis stages were derived from fibrosis test results.

Several types of fibrosis classifications are now available for non-invasive fibrosis tests, the most important of which is detailed *fibrosis class classification*. We developed a *fibrosis class classification *method specific to FibroMeter that defines six fibrosis classes based on F_M _classification [4]. Fibrotest and Fibroscan are the other tests with detailed *fibrosis class classifications*, but methodology details are lacking [5,6]. *Fibrosis class classification *is used in the commercial versions of these tests, especially Fibrotest and FibroMeter. Clinicians also use a simplified classification for Fibroscan [7]. However, the diagnostic characteristics, especially accuracy, of these classifications have not been thoroughly evaluated or validated. We recently performed a preliminary simple comparison in one population that suggested a large difference between two blood tests [8].

These non-invasive tests are used in clinical practice. In a previous study, we observed a poor agreement for liver biopsy by local pathologist compared to expert pathologist in clinical practice [9]. However, the accuracy of pathologists for fibrosis classification has never been compared with that of non-invasive tests in this setting.

Therefore, the main aim of the present study was to thoroughly evaluate the accuracies of the detailed *fibrosis class classifications *that have been developed for non-invasive fibrosis tests in patients with chronic HCV hepatitis based on liver biopsy as reference. The secondary aims were to compare these classification accuracies to that of histological staging by liver biopsy measured in clinical practice and to that of binary classification for significant fibrosis, which is the usual accuracy assessment of non-invasive tests. Finally, we evaluated the robustness of these accuracies in independent HCV populations.

## Methods

### Study design

We recruited different populations with liver biopsy to evaluate the different diagnostic means. Thus, population #1 provided different pathologist skill-levels and blood tests. The large population #2 included only blood tests. The more recent populations #3 and #4 included Fibroscan and blood tests. The four populations were separately analysed due to initial differences in study designs; this allowed us to evaluate accuracy robustness given these differences.

### Populations

Patients with chronic HCV hepatitis, liver biopsy, blood tests and available Fibroscan were consecutively recruited in different populations: #1 to #4 described in Table 1. Each population had different characteristics and fibrosis assessments. Inclusion and exclusion criteria are detailed in previous publications or below for new populations. Briefly, patients did not receive antiviral or known anti-fibrotic treatments. Liver biopsy, blood withdrawal and Fibroscan, when available, were performed within a maximum interval of 6 months. The study protocol conformed to the ethical guidelines of the current Declaration of Helsinki and was approved by local ethics committees. Patients gave written consent.

**Table 1:** Main characteristics of HCV populations.

Population #	Study name	Patients (n)	Liver biopsy length (mm)	Blood tests	FS	Metavir F prevalence (%)				
						
						0	1	2	3	4
1	Metavar 4	205	23 ± 7	x	-	4.4	46.3	29.8	14.1	5.4
2	Sniff 17	1056	21 ± 8	x	-	4.4	43.5	27.0	14.0	11.2
3	Fibrostar	458	25 ± 8	x	x	6.7	45.1	17.9	15.6	14.8
4	Vindiag 7	349	25 ± 9	x	x	1.4	30.7	35.5	20.6	11.7

x: test performed, FS: Fibroscan

*Population #1 *included 205 patients recruited from primary, secondary or tertiary care centres as detailed elsewhere [10] for a diagnostic study. Liver biopsy was read initially by a local (first line) pathologist, then independently by an expert from the Metavir group and finally by two other experts with a consensus reading in case of disagreement.

*Population #2 *included 1,056 patients provided by five centres participating in the Sniff 17 study [11]. Thus, individual patient data were available from five centres, independent for study design, patient recruitment, and blood marker determination. Blood and pathological determinations were not centralized. Pathological assessments were performed twice by the same pathologist in Grenoble, once in Bordeaux and once each by two pathologists in Angers, Tours and PACA region, with a common final reading in cases of disagreement.

*Population #3 *included 458 patients provided by 19 centres participating in the Fibrostar study [12]. Blood determination and liver interpretation were centralized. Liver specimens were read by two senior experts, one of whom was from the Metavir group.

*Population #4 *included 349 patients provided by three centres participating in the Vindiag 7 study (exploratory set) [13]. Blood and pathological (one senior expert in each centre) determinations were not centralized.

### Diagnostic means

Fibrosis was staged in liver biopsy according to Metavir staging [1] in all patients. This fibrosis stage classification was used as the reference for the calculation of accuracy. In population #1, where several readings were available, the consensus reading by two experts was the reference. "Expert pathologist" was defined as a senior pathologist specialized in hepatology. At least one expert pathologist was available in each study. Blood tests were determined in all studies; we only evaluated here those for which a detailed *fibrosis class classification *has been described, i.e., FibroMeter [14] (Biolivescale, Angers, France) and Fibrotest [5] (Biopredictive, Paris, France). Second generation FibroMeter (FibroMeter^2G^) [14], the most widely studied, and a recent third generation FibroMeter (FibroMeter^3G^) [8] were evaluated. Two studies also included Fibroscan (Echosens, Paris, France) as this technique has only been available since 2004; usual technical aspects have been described elsewhere [15]. All successful measurements of Fibroscan were included in the calculations.

### Fibrosis classifications

**Figure 1:**
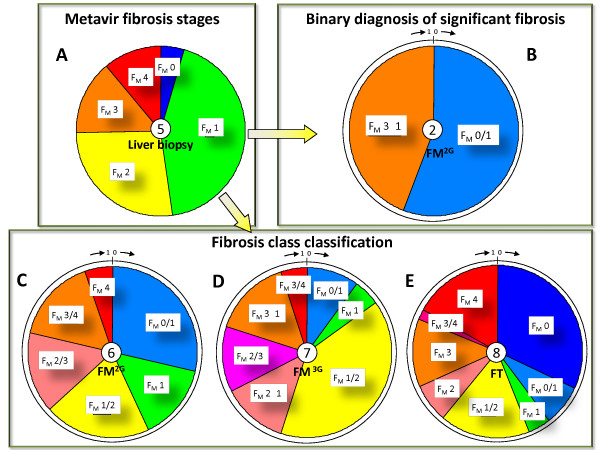
**Summary of different available fibrosis classifications in population #2**. Metavir stages by liver biopsy (A), significant fibrosis by FibroMeter^2G ^(FM) (B), *fibrosis class classification *by FibroMeter^2G ^(C) or FibroMeter^3G ^(D) or by Fibrotest (FT) (E). The central figure within the pie chart indicates the number of fibrosis classes. Sectors correspond to patient proportions. The figures in the external circle of panels reflect the values of blood test scores. F_M _denotes the Metavir fibrosis stages estimated by the classification.

We distinguished as fibrosis degrees the histological *fibrosis stages *and the *fibrosis classes *provided by non-invasive tests and including one or several *fibrosis stages*. Several fibrosis classifications were evaluated:

- The histological *fibrosis stage classification *into 5 F_M _stages (Figure 1a), as determined on a liver specimen by a pathologist. This was the reference for accuracy.

- The *binary diagnosis of significant fibrosis *(2 classes, Figure 1b) determined either on liver specimen or by the diagnostic cut-off in non-invasive tests. This is the usual diagnostic target of non-invasive tests and thus served as a comparator for the detailed classifications. Indeed, as it was expected that a more detailed classification would result in decreased accuracy, this binary accuracy allowed for the evaluation of the putative accuracy loss.

- The *fibrosis class classification *used in non-invasive tests, for which there are two main types:

• The classifications previously published for blood tests and Fibroscan. There are 6 classes for FibroMeter^2G ^(Figure 1c) [4], 7 for FibroMeter^3G ^(Figure 1d), 8 for Fibrotest (Figure 1e) [5] and 6 for Fibroscan [6]. The methodology for the development of FibroMeter^2G ^classification has been published [4]: briefly, the percentiles of blood test values were segmented into different intervals according to an absolute majority probability (p ≥ 0.75) for one or several F_M _stages (their number had to be ≤ 3). We developed an improved *fibrosis class classification *for FibroMeter^3G ^by using specific thresholds and changing slightly the fibrosis classes (Figure 1d). The optimization consisted in obtaining the best accuracy/precision ratio (number of Metavir fibrosis stages per fibrosis class of the non-invasive test).

• The classifications derived from the cumulated cut-offs calculated for different binary diagnostic targets, usually significant fibrosis and cirrhosis. Physicians normally use these kinds of classifications for the interpretation of Fibroscan results. This process results in a classification including 3 classes: F_M_0/1, F_M_2/3, and F_M_4. The cut-off for severe fibrosis (F_M_≥ 3) may also be used, resulting in a classification with 4 classes: F_M_0/1, F_M_2, F_M_3, and F_M_4. We used the diagnostic cut-offs calculated for HCV in the meta-analysis of Stebbing *et al *[7], giving the following three classes: < 8.44 kPa: F_M_0/1, ≥ 8.44 kPa and < 16.14 kPa: F_M_2/3, ≥ 16.14 kPa: F_M_4.

### Statistics

Data were reported according to STARD statements [16]. Quantitative variables were expressed as mean ± SD, unless otherwise specified. Metavir fibrosis staging was used either as a categorical variable or as a score (continuous variable) since we have shown a perfect linear correlation between Metavir fibrosis stages and fractal dimension of fibrosis which reflects quantitative architecture. For this reason, the results of *fibrosis class classification *were also evaluated as a score, e.g., F_M_3/4 class was noted as 3.5. This score was only used in the reflection evaluation of Metavir staging (see the fourth figure). Multivariate analyses were based on binary logistic regression. The performance of each test was mainly expressed by the accuracy (i.e., true positives and negatives or correct classification). The diagnostic cut-offs used for significant fibrosis were determined by a posteriori maximum Youden index (sensitivity + specificity - 1). Discrepancy between diagnostic means can be evaluated as grade or score. The grade rate shows details, especially the grade of significant discrepancy (≥ 2 F_M _stages). The *discrepancy score *took into account the magnitude of the error. This score was defined as follows: 0 for correct classification, then 1, 2, 3 or 4 as per the misclassification in F_M _stages between the liver specimen and the *fibrosis class classification *by the non-invasive test. For example, a patient with histological F_M_4 but classified as F_M_0/1 by blood test was scored 3. The mean score permits a comparison between blood tests. A low score means a low discrepancy magnitude. Statistical software programs were SPSS version 17.0 (SPSS Inc., Chicago, IL, USA) and SAS 9.1 (SAS Institute Inc., Cary, NC, USA).

## Results

### Liver biopsy

Population #1 was used to compare the accuracy of pathologists with different expertise levels or vs. blood tests. The prevalence of significant fibrosis was 49.3%.

#### Classification accuracy

*Metavir expert as reference - *The rates of correct classification for significant fibrosis and F_M _stages by local pathologists were, respectively: 77.1% and 52.2% (p < 10^-3 ^by McNemar test).

*Consensus reading as reference *- The rates of correct classification of the two single (local or expert) pathologists and two blood tests are listed in Table 2. Briefly, detailed fibrosis classifications could be ordered according to their accuracies as follows: FibroMeter^3G ^(89.0%) ≈ expert pathologist (82.2%) ≈ FibroMeter^2G ^(76.3%) > local pathologists (64.4%) > Fibrotest (34.3%). FibroMeter^2G ^was the only diagnostic method with no significant difference in correct classification rates between significant fibrosis diagnosis and *fibrosis class classification*. FibroMeter^3G ^was the only diagnostic method with a significant increase in correct classification rate of *fibrosis class classification *compared to significant fibrosis diagnosis.

**Table 2:** Rates of correct classification (%, bold characters) as a function of diagnostic means in population #1.

	Significant fibrosis (F_M _≥ 2)	**Fibrosis degree **^**a**^	**p **^**b**^
Local pathologists	**85.9**	**64.4**	< 10^-3^
Expert pathologist	**91.4**	**82.2**	< 10^-3^
Fibrotest (FT)	**74.2**	**34.3**	< 10^-3^
FibroMeter^2G ^(FM^2G^)	**75.3**	**76.3**	0.860
FibroMeter^3G ^(FM^3G^)	**75.5**	**89.0**	< 10^-3^
Comparison ^b^:	p	p	-
All	< 10^-3^	< 10^-3^	-
Local pathologist vs. expert	0.184	< 10^-3^	-
Local pathologist vs. FT	0.003	< 10^-3^	-
Local pathologist vs. FM^2G^	0.005	0.007	-
Local pathologist vs. FM^3G^	0.004	< 10^-3^	-
Expert pathologist vs. FT	< 10^-3^	< 10^-3^	-
Expert pathologist vs. FM^2G^	< 10^-3^	0.092	-
Expert pathologist vs. FM^3G^	< 10^-3^	0.126	-
FT vs. FM^2G^	0.839	< 10^-3^	-
FT vs. FM^3G^	0.878	< 10^-3^	-
FM^2G ^vs. FM^3G^	1	< 10^-3^	-

The reference is consensus reading of liver biopsy.

^a ^Metavir staging for pathologist or *fibrosis class classification *for blood tests

^b ^By McNemar test (pair) or Friedman test (all)

#### Discrepancy

The *discrepancy scores *were significantly different between pathologists: local vs. expert: 0.55 ± 0.63, local vs. consensus: 0.40 ± 0.58, expert vs. consensus: 0.17 ± 0.38 (p < 10^-3 ^by paired Friedman test). In addition, the proportions of significant discrepancies (≥ 2 F_M _stages) were significantly different: local vs. expert: 7.3%, local vs. consensus: 4.9%, expert vs. consensus: 0% (p < 10^-3 ^by paired Cochran test).

When considering consensus reading by experts as reference, the *discrepancy score *of FibroMeter^2G ^was significantly lower than that of local pathologists (p = 0.043) but significantly higher than that of the expert pathologist (p = 0.006, Table 3). This latter was not significantly different from that of FibroMeter^3G ^(p = 0.077). The *discrepancy score *of Fibrotest was significantly higher than that of local or expert pathologists (p < 10^-3^). In addition, the proportions of significant discrepancies were very different: FibroMeter^3G ^< FibroMeter^2G ^< Fibrotest (p < 10^-3 ^by paired Cochran test, Table 3).

**Table 3:** Discrepancy against a diagnostic reference.

	Discrepancy score				Significant discrepancies (%)			
		
Population #	**1**^**a**^	2	3	4	**1**^**a**^	2	3	4
Local pathologist	0.40 ± 0.58	-	-	-	4.9	-	-	-
Expert pathologist	0.17 ± 0.38	-	-	-	0.0	-	-	-
Fibrotest	0.86 ± 0.77	0.84 ± 0.80	0.86 ± 0.93	0.92 ± 0.82	17.2	18.2	21.3	22.2
FibroMeter^2G^	0.30 ± 0.58	0.30 ± 0.55	0.36 ± 0.62	0.38 ± 0.61	5.6	4.6	5.7	6.0
FibroMeter^3G^	0.11 ± 0.33	0.14 ± 0.37	0.23 ± 0.44	0.17 ± 0.40	0.5	0.7	0.9	0.9
Fibroscan	-	-	0.50 ± 0.79	0.64 ± 0.74	-	-	12.9	12.3
p ^b^	< 10^-3^	< 10^-3^	< 10^-3^	< 10^-3^	< 10^-3^	< 10^-3^	< 10^-3^	< 10^-3^

Discrepancy score and significant discrepancies (≥ 2 F_M _stages) with liver biopsy results as a function of *fibrosis classifications *by pathologists, blood tests or Fibroscan according to the 4 populations.

^a ^The reference is consensus reading of liver biopsy

^b ^by paired Cochran or Friedman test

### Blood tests

Results are detailed in population #2 since it was the largest (1,056 patients) for blood tests.

#### Classification accuracy

The accuracy of *fibrosis class classification *by FibroMeter^2G^, FibroMeter^3G ^and Fibrotest have been presented elsewhere [8] and will discussed further on.

#### Discrepancy

**Figure 2:**
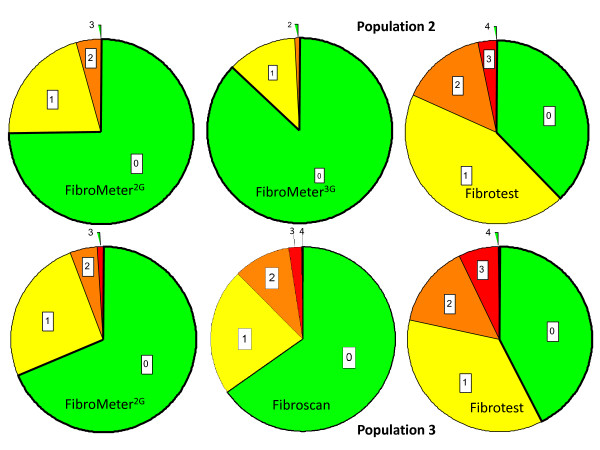
**Rates of discrepancy grade of *fibrosis class classifications *by diagnostic tests in populations #2 (top) or #3 (bottom)**. The figure indicates the difference in the number of fibrosis stage(s) between the blood test and liver biopsy. Thus, the grade 0 (green pie sector) indicates agreement with liver biopsy.

The *discrepancy scores *were significantly different between FibroMeter^2G ^and FibroMeter^3G ^(p < 10^-3^) or Fibrotest (p < 10^-3^, Table 3). Details on *discrepancy grade *are shown in Figure 2. In addition, the proportion of significant discrepancies with FibroMeter^2G ^or FibroMeter^3G ^was significantly lower than with Fibrotest (p < 10^-3 ^by McNemar test, Table 3).

### Elastometry

Populations #3 and #4 were used to compare elastometry by Fibroscan and blood tests.

#### Classification accuracy

In population #3 (and #4), the accuracies of the *fibrosis class classifications *were 42.5% (33.5%) for Fibrotest, 64.9% (50.7%) for Fibroscan, 68.7% (68.2%) for FibroMeter^2G^, and 77.1% (83.4%) for FibroMeter^3G^, p < 10^-3 ^(p < 10^-3^) between non-invasive tests (Table 4).

**Table 4:** Rates of correct classification by non-invasive means (%, bold characters) as a function of fibrosis classification in populations #3 and #4.

	Population #3			Population #4		
		
	Significant fibrosis (F_M _≥ 2)	Fibrosis class classification	**p **^**a**^	Significant fibrosis (F_M _≥ 2)	Fibrosis class classification	**p**^**a**^
Fibrotest (FT)	**71.3**	**42.5**	< 10^-3^	**75.2**	**33.5**	< 10^-3^
FibroMeter^2G ^(FM^2G^)	**75.2**	**68.7**	0.001	**77.7**	**68.2**	< 10^-3^
FibroMeter^3G ^(FM^3G^)	**74.0**	**77.1**	0.255	**76.8**	**83.4**	0.011
Fibroscan (FS)	**73.7**	**64.9**	< 10^-3^	**75.2**	**50.7 (52.8) ^b^**	< 10^-3 ^(< 10^-3^)
Comparison ^a^:	p	p	-	p	p	-
All	0.644	< 10^-3^	-	< 10^-3^	< 10^-3^	-
FT vs. FM^2G^	0.101	< 10^-3^	-	0.314	< 10^-3^	-
FT vs. FM^3G^	0.064	< 10^-3^	-	0.504	< 10^-3^	-
FT vs. FS	0.344	< 10^-3^	-	1	< 10^-3 ^(< 10^-3^)	-
FM^2G ^vs. FM^3G^	1	< 10^-3^	-	0.549	< 10^-3^	-
FM^2G ^vs. FS	0.549	0.121	-	0.497	< 10^-3 ^(< 10^-3^)	-
FM^3G ^vs. FS	1	< 10^-3^	-	0.699	< 10^-3^	-

^a ^By McNemar test (pair) or Friedman test (all)

^b ^Classification into 6 [6] or 3 [7] classes in parentheses

#### Discrepancy

**Figure 3:**
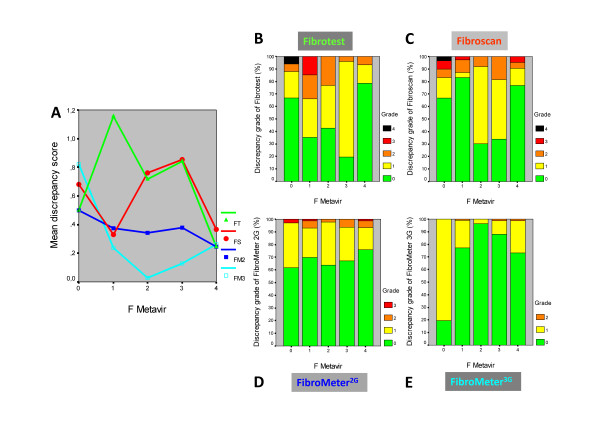
**Discrepancy between *fibrosis class classifications *by non-invasive tests and liver biopsy staging**. Results (Y axis) are expressed as a function of Metavir fibrosis (F) stage (X axis) in population #3. The left panel **A **indicates the mean score. The right panels show the details of discrepancy grades for each diagnostic test: Fibrotest (**B**), Fibroscan (**C**), FibroMeter^2G ^(**D**) and FibroMeter^3G ^(**E**). The grade indicates the difference in the number of fibrosis stage(s) between the blood test and liver biopsy. FT: Fibrotest, FS: Fibroscan, FM2: FibroMeter^2G^, FM3: FibroMeter^3G^.

In population #3 and #4, the discrepancy scores were significantly different: FibroMeter^3G ^< FibroMeter^2G ^< Fibroscan < Fibrotest (p < 10^-3 ^by Friedman test in each population, Table 3), with only FibroMeter^2G ^offering a homogeneous score among F_M _stages (Figure 3). Details on *discrepancy grade *are shown in Figure 2. The proportions of significant discrepancies were also significantly different among fibrosis tests (p < 10^-3 ^by Cochran test in each population, Table 3).

### Reflection of histological stages by classifications

**Figure 4:**
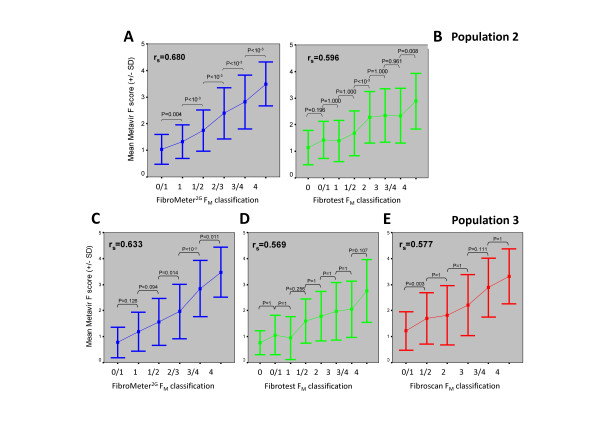
**Mean Metavir fibrosis score as a function of Metavir-based *fibrosis class classifications***. Results (± standard deviation, Y axis) are expressed as a function of classifications (X axis) for: FibroMeter^2G ^(panels A and C, 6 classes), Fibrotest (panels B and D, 8 classes) or Fibroscan (panel E, 6 classes) in populations #2 (top) or #3 (bottom). P by weighted Bonferroni test. The global relationship is indicated by Spearman's correlation coefficient (r_s_).

In population #2, the *fibrosis class classification *of FibroMeter^2G ^(expressed as score) was more closely correlated with F_M _score than that of Fibrotest (Figure 4a/b). By ANOVA, the mean F_M _score was significantly different as a function of *fibrosis class classification *of FibroMeter^2G ^(F = 188, p < 10^-4^) and Fibrotest (F = 83, p < 10^-4^). However, the post hoc comparison (by weighted Bonferroni test) showed highly significant differences between each pair of fibrosis classes for FibroMeter^2G^, whereas this was not observed between several pairs of contiguous classes of Fibrotest (Figure 4a/b).

Results in population #3 were similar to those observed in population #2: significant discrimination between most contiguous fibrosis classes by FibroMeter^2G ^and any significant discrimination by Fibrotest (Figure 4c/d). Fibroscan classification was poorly discriminating between contiguous classes (Figure 4e).

**Figure 5:**
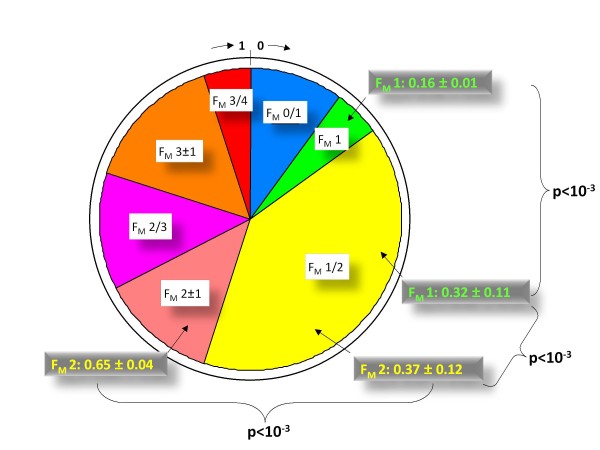
**Meaning of blood test score (in grey rectangles) in different Metavir fibrosis (F_M_) stages within the same class of *fibrosis class classification***. Example of F_M_2 and F_M_1 stages in FibroMeter^3G ^in population #2. Sectors correspond to patient proportions. The figures on the top of the external circle reflect the values (mean ± SD) of the blood test score for a single F_M _stage. The significant difference between F_M _stages of contiguous classes was mathematically expected contrary to that observed within a single class.

The *fibrosis class classification *might offer some degree of imprecision in the classes including at least two F_M _stages. Therefore, we evaluated the meaning of test score within the largest class observed, i.e., F_M_1/2 class with FibroMeter^3G ^in population #2 (Figure 5). In this class, FibroMeter^3G ^score was 0.32 ± 0.11 in F_M_1 vs. 0.37 ± 0.12 in F_M_2 (p < 10^-3^).

## Discussion

### Liver biopsy

In this study, we have shown that the *fibrosis class classification *of an accurate blood test like FibroMeter^2G ^provides better accuracy than Metavir staging by local pathologists, which reflects clinical practice. Additionally, its accuracy was not significantly different from that of Metavir staging by a senior expert of the Metavir group. Surprisingly, *fibrosis class classification *of FibroMeter^3G ^provided a non-significantly higher accuracy than that of the senior expert of the Metavir group. This can be attributed to the poor inter-observer agreement of liver interpretation for fibrosis staging in clinical practice [9].

These results nonetheless deserve some comments. First, the accuracy of liver biopsy was significantly superior to that of the best performing non-invasive test when the diagnostic target was binary, such as significant fibrosis. In other words, the development of detailed *fibrosis class classifications *derived from F_M _stages compensated for the lesser performance of non-invasive tests in binary diagnostic targets, as observed in the literature and in the present study. Second, *fibrosis class classifications *of non-invasive tests seem less precise at first glance; we discuss this important characteristic further on. Third, this study underlines the issue of reference, as an expert from the Metavir group underperformed the consensus reading considered as reference in the present study. Thus, who, or what, should be used as a reference? We have already observed that a consensus reading improved reproducibility and thus could be considered as a reference [9]. However, we do not know if a panel reading would be a more reliable reference. Liver biopsy does have innate limits, such as sampling error and sample size effect, which surpass those of liver interpretation. Indeed, two studies have recently shown that blood tests for liver fibrosis were better prognosis predictors than histological staging [17,18].

### Non-invasive tests

Liver biopsy was used as the best standard [19]. Despite its limits, it can be considered as a good reference for the comparison between non-invasive tests since there are no data to consider that the biopsy error was not systematic (i.e., different between tests). In other words, the accuracy of non-invasive tests is probably underestimated but not their comparison. The results of the different populations are summarized in table 5. The accuracies of *fibrosis class classifications *were different among non-invasive tests in the present study in the following order: FibroMeter^3G ^> FibroMeter^2G ^> Fibroscan > Fibrotest. It should be underlined that these differences were observed in several independent populations. In addition, from one study to another, the rank of accuracy between tests was very reproducible. Thus, the present results are robust. It should also be noted that the authors of a recent study using a quite different methodology in a small series (four patients) observed an accuracy of less than 25% with the *fibrosis stage classification *of Fibrotest [20]. How thus can one explain this apparent discrepancy between the close accuracies of non-invasive tests for the usual binary diagnostic targets such as significant fibrosis, and the dissimilar accuracies in their *fibrosis class classifications*? First, a single binary diagnostic target necessarily (mathematically) includes fewer sources of errors than a multiple-stage classification. Second, the statistical methods used to develop the *fibrosis class classifications *have to be considered. We developed a new statistical method for the development of a *fibrosis class classification *[4]. Thus, we obtained a *fibrosis class classification *with FibroMeter^2G ^that included 6 classes, each one comprising only one or two Metavir fibrosis stage(s). It should be noted that the *fibrosis class classifications *of Fibrotest or Fibroscan have been reported but the statistical methodology used to establish them was not described [5,6], nor their accuracy. The method used for three stage classification of Fibroscan accumulates the misclassification rates of each diagnostic cut-off. We used the cut-offs of Stebbing et al since their study was a large recent meta-analysis restricted to HCV. The method of *fibrosis class classification *that we developed for FibroMeter^2G ^[4] was validated in the present study by the reproducible accuracy measured in several independent large populations. Thus, before using a non-invasive test in clinical practice, it seems important to verify the statistical methodology behind the construct and its accuracy.

**Table 5:** Summary of correct classification rates (%) and score/grade discrepancy (2 bottom lines).

	Liver biopsy		FibroMeter								Fibrotest				Fibroscan	
				
			2G				3G									
										
Population #	1	1	1	2	3	4	1	2	3	4	1	2	3	4	3	4
Pathologist	Local ^a^	Expert	-	-	-	-	-	-	-	-	-	-	-	-	-	-
Metavir F_M _staging	52.2/64.4	82.2	-	-	-	-	-	-	-	-	-	-	-	-	-	-
Binary diagnosis ^b^	77.1/85.9	91.4	75.3	78.1*	75.2	77.7	75.5	77.9*	74.0	76.8	74.2	74.5*	71.3	75.2	73.7	75.2
Fibrosis class classification ^c^	-	-	76.3	74.9*	68.7	68.2	89.0	86.9*	77.1	83.4	34.3	37.9*	42.5	33.5	64.9	50.7

Discrepancy score ^d^	0.55/0.40	0.17	0.30	0.30	0.36	0.38	0.11	0.14	0.23	0.17	0.86	0.84	0.86	0.92	0.50	0.64
Significant discrepancy (%) ^e^	7.3/4.9	0.0	5.6	4.6	5.7	6.0	0.5	0.7	0.9	0.9	17.2	18.2	21.3	22.2	12.9	12.3

Results are presented according to different classifications and diagnostic means in the 4 populations with hepatitis C.

^a ^The first figure refers to the expert as reference and the second to the consensus reading as reference

^b ^for significant fibrosis; results indicated with * were provided by a previous study [8]

^c ^by blood test; results indicated with * were provided by a previous study [8]

^d ^Mean

^e ^≥ 2 F_M _stage

The present results indicate that the FibroMeter classification is robust, as its precision was expanded from 2 for significant fibrosis to 6 or 7 fibrosis classes at the expense of only a 4% relative decrease in FibroMeter^2G ^accuracy or a 12% relative increase in FibroMeter^3G ^accuracy (87% in the largest series) [8]. It should be noted that the accuracy/precision ratio was optimized only for FibroMeter^3G ^[8] but this optimization could also be applied to FibroMeter^2G^. This contrasts with Fibrotest, which displayed a 49% relative decrease in accuracy in the largest series between the binary diagnosis and its 8-class fibrosis classification [8]. In addition, the FibroMeter^2G ^*fibrosis class classification *was more discriminant than those of Fibrotest or Fibroscan in distinguishing fibrosis classes, especially two successive classes (Figure 4). It has been suggested that the maximal theoretical accuracy may be around 90%, considering the limits of liver biopsy as a reference [21].

The discrepancy level between *fibrosis class classifications *of non-invasive tests and Metavir stages was reflected by the discrepancy score and the proportion of significant discrepancy (≥ 2 F_M_), which markedly varied among tests in the present study. FibroMeter^2G ^and even FibroMeter^3G ^provided a significantly lower discrepancy score than Fibrotest or Fibroscan in all study populations.

### Best classifications for clinical use

**Figure 6:**
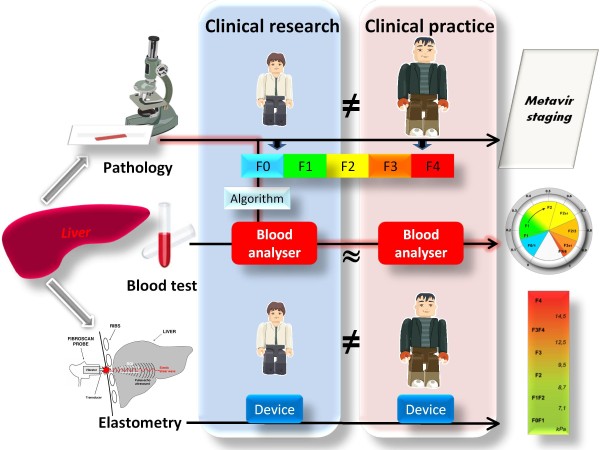
**Schematic reliability of diagnostic means**. In clinical practice, a blood test is more reliable than liver pathology since the blood test is based on an algorithm that was calculated with expert pathologist as reference (black arrow with red background). There is little procedure variability for blood tests due to excellent interlaboratory reproducibility, contrary to the large inter-observer disagreement for liver pathology and, to a lesser degree, for elastometry. The size of observers is proportional to published observer variability.

The accuracy (correct classification in the whole population) of binary diagnosis was superior or equal to that of *fibrosis class classification *except for FibroMeter^3G^. However, the level of classification precision (less fibrosis stages per class) also has to be examined. When the ratio between accuracy and precision is considered, *fibrosis class classification *seems to provide the best performance. Finally, the *fibrosis class classification *of FibroMeter^2G ^had a significantly higher correct classification (qualitative accuracy descriptor) and a significantly lower discrepancy level (quantitative accuracy descriptor better reflecting disagreement than the former) compared to local pathologists. In addition, FibroMeter^3G ^compared favourably with expert pathologist for those characteristics. This better accuracy for the *fibrosis class classification *of FibroMeters as compared to liver biopsy would seem to provide a strong argument for their use in clinical practice despite their lesser precision. In other words, FibroMeters had fewer errors than liver biopsy interpretation in clinical practice. Figure 6 also shows that a blood test has a robust diagnostic reproducibility in clinical practice, compared to other diagnostic means. However, this issue of precision can be refined.

### Interpreting classifications

Based on F_M _stages, *fibrosis class classifications *provide multiple classes of F_M _stages according to blood test values [4]. Thus, FibroMeter^2G ^*fibrosis class classification *provided the following new classes: F_M_0/1, F_M_1, F_M_1/2, F_M_2/3, F_M_3/4 and F_M_4. These correspond to the following FibroMeter fibrosis stages expressed in single Metavir score: F_M_0.5, F_M_1, F_M_1.5, F_M_2.5, F_M_3.5, and F_M_4. They can furthermore be translated into the following new FibroMeter^2G ^fibrosis (F_FM_) stages: F_FM_0, F_FM_1, F_FM_2, F_FM_3, F_FM_4 and F_FM_5. This last classification assumes that there is less error with non-invasive tests than with liver biopsy, as suggested by several studies [22,23]. Therefore, the interest of these new classifications, based on "blood" fibrosis stages, has to be tested independently of their native histological reference by using clinical events as an endpoint. This could be accomplished through a prognostic study as previously done for blood tests used as scores [17,18] from which classifications are derived. Finally, it should be noted that within the largest FibroMeter^3G ^fibrosis class, the score progression of blood test well reflected the histological progression (Figure 5).

### Limits

The prevalence of significant fibrosis in the four populations was close to that (48%) of a reference population of 33,121 patients with HCV and liver biopsy [24]. The studies including Fibroscan were not based on an intention-to-diagnose analysis since unsuccessful measurements were not included. This would decrease the accuracy by about 5% as already shown in another study [25] but not modify the hierarchy of tests regarding accuracy. It should be underlined that liver biopsy has other indications than liver fibrosis.

## Conclusions

Liver biopsy is useful for fibrosis staging if the reading is performed by an expert, or even better, by consensus including preferably at least one expert. Accuracies varied very significantly between the *fibrosis class classifications *of the non-invasive tests. With the best performing test, this classification has two advantages: increased precision and accuracy compared to a binary diagnosis of significant fibrosis; and similar or higher accuracy when compared to histological staging performed in clinical practice conditions. However, the accuracy/precision ratio was higher with Metavir staging by definition, since this was the reference. These results, observed in hepatitis C, should be evaluated in other causes (see Additional File 1). Finally, the classification of a good-performing test permits the evaluation of the degree of fibrosis in settings where liver biopsy is not available or feasible, such as in epidemiological studies.

## Abbreviations

F_M_: fibrosis in Metavir staging; HCV: hepatitis C virus.

## Competing interests

Paul Calès, Isabelle Fouchard Hubert and Frédéric Oberti have stock ownership in BioLiveScale Inc. BioLiveScale has a license for FibroMeter from Angers University. Other authors: no conflicts of interest to declare.

## Authors' contributions

JB: planning and conducting the study, collecting and interpreting data, drafting the manuscript, read and approved the final manuscript. SB: statistical analysis, read and approved the final manuscript. FO: collecting and interpreting data, read and approved the final manuscript. YG: collecting and interpreting data (biochemical analysis), read and approved the final manuscript. IFH: collecting and interpreting data, read and approved the final manuscript. MCR: collecting and interpreting data (pathological analysis), read and approved the final manuscript. JPZ: planning and conducting the Fibrostar study, collecting and interpreting data, read and approved the final manuscript. PC: planning and conducting the study, collecting and interpreting data, drafting the manuscript, read and approved the final manuscript

## Pre-publication history

The pre-publication history for this paper can be accessed here:

http://www.biomedcentral.com/1471-230X/11/132/prepub

## Supplementary Material

Additional file 1 **Supplementary results**. We present a glossary of fibrosis classifications in Additional File 1, **Table S1**. We also present here detailed results on score and grade of discrepancy, the reflection of histological stages by classifications and performance profiles of blood tests as well as the accuracies of *fibrosis class classifications *in causes of chronic liver disease other than HVC. Click here for file

## References

[B1] Intraobserver and interobserver variations in liver biopsy interpretation in patients with chronic hepatitis C. The French METAVIR Cooperative Study Group Hepatology 1994 20 1 Pt 1 15 20 8020885

[B2] Ishak K Baptista A Bianchi L Callea F De Groote J Gudat F Denk H Desmet V Korb G MacSween RN Histological grading and staging of chronic hepatitis J Hepatol 1995 22 6 696 699 10.1016/0168-8278(95)80226-6 7560864

[B3] Everhart JE Wright EC Goodman ZD Dienstag JL Hoefs JC Kleiner DE Ghany MG Mills AS Nash SR Govindarajan S Prognostic value of Ishak fibrosis stage: findings from the hepatitis C antiviral long-term treatment against cirrhosis trial Hepatology 2010 51 2 585 594 10.1002/hep.23315 20101752 PMC3814134

[B4] Leroy V Halfon P Bacq Y Boursier J Rousselet MC Bourliere M de Muret A Sturm N Hunault G Penaranda G Diagnostic accuracy, reproducibility and robustness of fibrosis blood tests in chronic hepatitis C: a meta-analysis with individual data Clin Biochem 2008 41 16-17 1368 1376 10.1016/j.clinbiochem.2008.06.020 18655779

[B5] Poynard T Imbert-Bismut F Munteanu M Messous D Myers RP Thabut D Ratziu V Mercadier A Benhamou Y Hainque B Overview of the diagnostic value of biochemical markers of liver fibrosis (FibroTest, HCV FibroSure) and necrosis (ActiTest) in patients with chronic hepatitis C Comp Hepatol 2004 3 1 8 10.1186/1476-5926-3-8 15387887 PMC522750

[B6] de Ledinghen V Vergniol J Transient elastography (FibroScan) Gastroenterol Clin Biol 2008 32 6 Suppl 1 58 67 18973847 10.1016/S0399-8320(08)73994-0

[B7] Stebbing J Farouk L Panos G Anderson M Jiao LR Mandalia S Bower M Gazzard B Nelson M A meta-analysis of transient elastography for the detection of hepatic fibrosis J Clin Gastroenterol 2010 44 3 214 219 10.1097/MCG.0b013e3181b4af1f 19745758

[B8] Cales P Boursier J Bertrais S Oberti F Gallois Y Fouchard-Hubert I Dib N Zarski JP Rousselet MC Optimization and robustness of blood tests for liver fibrosis and cirrhosis Clin Biochem 2010 43 16-17 1315 1322 10.1016/j.clinbiochem.2010.08.010 20713037

[B9] Rousselet MC Michalak S Dupre F Croue A Bedossa P Saint-Andre JP Cales P Sources of variability in histological scoring of chronic viral hepatitis Hepatology 2005 41 2 257 264 10.1002/hep.20535 15660389

[B10] Halfon P Bacq Y De Muret A Penaranda G Bourliere M Ouzan D Tran A Botta D Renou C Brechot MC Comparison of test performance profile for blood tests of liver fibrosis in chronic hepatitis C J Hepatol 2007 46 3 395 402 10.1016/j.jhep.2006.09.020 17156890

[B11] Cales P de Ledinghen V Halfon P Bacq Y Leroy V Boursier J Foucher J Bourliere M de Muret A Sturm N Evaluating the accuracy and increasing the reliable diagnosis rate of blood tests for liver fibrosis in chronic hepatitis C Liver Int 2008 28 10 1352 1362 10.1111/j.1478-3231.2008.01789.x 18492022 PMC2711538

[B12] Zarski JP Sturm N Guechot J Paris A Zafrani ES Asselah T Boisson RC Bosson JL Guyader D Renversez JC Comparison of nine blood tests and transient elastography for liver fibrosis in chronic hepatitis C: The ANRS HCEP-23 study J Hepatol 2011 10.1016/j.jhep.2011.05.024 21781944

[B13] Boursier J de Ledinghen V Zarski JP Rousselet MC Sturm N Foucher J Leroy V Fouchard-Hubert I Bertrais S Gallois Y A new combination of blood test and fibroscan for accurate non-invasive diagnosis of liver fibrosis stages in chronic hepatitis C Am J Gastroenterol 2011 106 7 1255 1263 10.1038/ajg.2011.100 21468012

[B14] Cales P Oberti F Michalak S Hubert-Fouchard I Rousselet MC Konate A Gallois Y Ternisien C Chevailler A Lunel F A novel panel of blood markers to assess the degree of liver fibrosis Hepatology 2005 42 6 1373 1381 10.1002/hep.20935 16317693

[B15] Boursier J Vergniol J Sawadogo A Dakka T Michalak S Gallois Y Le Tallec V Oberti F Fouchard-Hubert I Dib N The combination of a blood test and Fibroscan improves the non-invasive diagnosis of liver fibrosis Liver Int 2009 29 10 1507 1515 10.1111/j.1478-3231.2009.02101.x 19725892

[B16] Bossuyt PM Reitsma JB Bruns DE Gatsonis CA Glasziou PP Irwig LM Moher D Rennie D de Vet HCW Lijmer JG The STARD statement for reporting studies of diagnostic acuracy: explanation and elaboration Clin Chem 2003 49 1 7 18 10.1373/49.1.7 12507954

[B17] Mayo MJ Parkes J Adams-Huet B Combes B Mills AS Markin RS Rubin R Wheeler D Contos M West AB Prediction of clinical outcomes in primary biliary cirrhosis by serum enhanced liver fibrosis assay Hepatology 2008 48 5 1549 1557 10.1002/hep.22517 18846542 PMC2597274

[B18] Naveau S Gaude G Asnacios A Agostini H Abella A Barri-Ova N Dauvois B Prevot S Ngo Y Munteanu M Diagnostic and prognostic values of noninvasive biomarkers of fibrosis in patients with alcoholic liver disease Hepatology 2009 49 1 97 105 10.1002/hep.22576 19053048

[B19] Bedossa P Carrat F Liver biopsy: the best, not the gold standard J Hepatol 2009 50 1 1 3 19017551 10.1016/j.jhep.2008.10.014

[B20] Gressner OA Beer N Jodlowski A Gressner AM Impact of quality control accepted inter-laboratory variations on calculated Fibrotest/Actitest scores for the non-invasive biochemical assessment of liver fibrosis Clin Chim Acta 2009 409 1-2 90 95 10.1016/j.cca.2009.09.005 19748500

[B21] Mehta SH Lau B Afdhal NH Thomas DL Exceeding the limits of liver histology markers J Hepatol 2009 50 1 36 41 10.1016/j.jhep.2008.07.039 19012989 PMC2637134

[B22] Poynard T Munteanu M Imbert-Bismut F Charlotte F Thabut D Le Calvez S Messous D Thibault V Benhamou Y Moussalli J Prospective analysis of discordant results between biochemical markers and biopsy in patients with chronic hepatitis C Clin Chem 2004 50 8 1344 1355 10.1373/clinchem.2004.032227 15192028

[B23] Halfon P Bourliere M Deydier R Botta-Fridlund D Renou C Tran A Portal I Allemand I Bertrand JJ Rosenthal-Allieri A Independent prospective multicenter validation of biochemical markers (fibrotest-actitest) for the prediction of liver fibrosis and activity in patients with chronic hepatitis C: the fibropaca study Am J Gastroenterol 2006 101 3 547 555 10.1111/j.1572-0241.2006.00411.x 16542291

[B24] Thein HH Yi Q Dore GJ Krahn MD Estimation of stage-specific fibrosis progression rates in chronic hepatitis C virus infection: a meta-analysis and meta-regression Hepatology 2008 48 2 418 431 10.1002/hep.22375 18563841

[B25] Boursier J Isselin G Fouchard-Hubert I Oberti F Dib N Lebigot J Bertrais S Gallois Y Cales P Aube C Acoustic radiation force impulse: a new ultrasonographic technology for the widespread noninvasive diagnosis of liver fibrosis Eur J Gastroenterol Hepatol 2010 22 9 1074 1084 10.1097/MEG.0b013e328339e0a1 20440210

