# Supplementary material for: Comparison of accuracy of fibrosis degree classifications by liver biopsy and non-invasive tests in chronic hepatitis C
Source: BMC Gastroenterol. 2011 Nov 30;11:132. doi: 10.1186/1471-230X-11-132 (PMC3247188; doi:10.1186/1471-230X-11-132)
Supplement: Additional file 1 — Supplementary results. We present a glossary of fibrosis classifications in Additional File 1, Table S1. We also present here detailed results on score and grade of discrepancy, the reflection of histological stages by classifications and performance profiles of blood tests as well as the accuracies of fibrosis class classifications in causes of chronic liver disease other than HVC. [file 1471-230X-11-132-S1.DOC]

**AdditIonal File 1**

**TITLE: Supplementary results**

**DescriPTION:** We present a glossary of fibrosis classifications in **Additional File 1, Table S1.** We also present here detailed results on score and grade of discrepancy, the reflection of histological stages by classifications and performance profiles of blood tests as well as the accuracies of *fibrosis class classifications* in causes of chronic liver disease other than HVC.

**Additional populations**

Populations #6 to #8 included patients with other causes of chronic liver disease, i.e., non-alcoholic fatty liver disease (NAFLD), alcoholic liver disease (ALD), and HCV/HIV co-infection (**Additional File 1, Table S2**). The use of the Metavir system for the determination of blood test for liver fibrosis has been validated in NAFLD and ALD .

*Population* #*6* included 444 patients with HCV/HIV co-infection provided by three cohorts . The main results were calculated in the derivation population including 176 patients recruited in 5 centres. The validation population was provided by the ANRS cohorts HC02 Ribavic and Co13 Hepavih .

*Population* #*7*  included 235 patients with NAFLD provided by two centres .

*Population* #*8*  included 241 patients with ALD provided by two centres .

**Statistics**

The correct classification rate of blood tests for significant fibrosis, also called *test performance profile* , was calculated in each Metavir F stage(s).

**Results**

***Classification agreement between expert pathologist and other evaluations***

Accuracies were listed in the main text. Detailed results are provided in **Additional File 1, Figure S1**.

***Performance profiles***

The details of correctly classified patients as a function of Metavir fibrosis stages showed different *performance profiles* for diagnostic tests (**Additional File 1, Figure S2**). Thus, rates of correctly classified patients had the following coefficients of variation among classes of *fibrosis class classifications*: FibroMeter^2G^: 23.1%, Fibrotest: 57.5%, Fibroscan: 49.6% (**Additional File 1, Figure S2a-c**).

The classical *performance profile* (accuracy for significant fibrosis as a function of F_M_ stages) showed a V pattern for each test as expected (**Additional File 1, Figure S2d:** dashed lines). The inconvenience of this V pattern (high proportion of misclassified patients in middle stages) was eliminated only by the *fibrosis class classification* of FibroMeter^2G^ (**Additional File 1, Figure S2d:** continuous lines). Finally, the mean accuracy of *fibrosis class classifications* by non-invasive tests was compared as a function of F_M_ stages: accuracy was stable for FibroMeter^2G^ classification (p=0.422 by ANOVA), i.e., displaying a homogeneous profile, but significantly varied for Fibrotest (p<10^-3^) and Fibroscan (p<10^-3^) (**Additional File 1, Figure S2d**: continuous lines).

***Other causes***

Results of correct *fibrosis class classification* by specific FibroMeters were available in population #6 (HCV/HIV): 81.6%, #7 (NAFLD): 89.8% and #8 (ALD): 77.3% with details reported in **Additional File 1, Table S3**. Other non-invasive tests, non-specific for cause, were not available.

**Discussion**

In causes other than HVC, the FibroMeter *fibrosis class classifications* were specific since blood tests were specific. A decrease in accuracy for *fibrosis class classification* compared to binary diagnosis was only observed with ALD. As these results were observed in pivotal studies, these *fibrosis class classifications* of FibroMeter (and other tests) should be validated in independent populations.

**References**

1. Cales P, Laine F, Boursier J, Deugnier Y, Moal V, Oberti F, Hunault G, Rousselet MC, Hubert I, Laafi J *et al*: **Comparison of blood tests for liver fibrosis specific or not to NAFLD**. *J Hepatol* 2009, **50**(1):165-173.

2. Cales P, Boursier J, Chaigneau J, Laine F, Sandrini J, Michalak S, Hubert I, Dib N, Oberti F, Bertrais S *et al*: **Diagnosis of different liver fibrosis characteristics by blood tests in non-alcoholic fatty liver disease**. *Liver Int* 2010, **30**(9):1346-1354.

3. Michalak S, Rousselet MC, Bedossa P, Pilette C, Chappard D, Oberti F, Gallois Y, Cales P: **Respective roles of porto-septal fibrosis and centrilobular fibrosis in alcoholic liver disease**. *J Pathol* 2003, **201**(1):55-62.

4. Calès P, Halfon P, Batisse D, Carrat F, Perré P, Penaranda G, Guyader D, d'Alteroche L, Fouchard-Hubert I, Michelet C *et al*: **Comparison of liver fibrosis blood tests developed for HCV with new specific tests in HIV/HCV co-infection** *J Hepatol* 2010, **52**:238-244.

5. Cacoub P, Carrat F, Bedossa P, Lambert J, Penaranda G, Perronne C, Pol S, Halfon P: **Comparison of non-invasive liver fibrosis biomarkers in HIV/HCV co-infected patients: The fibrovic study - ANRS HC02**. *J Hepatol* 2008, **48**(5):765-773.

6. Michel L, Villes V, Dabis F, Spire B, Winnock M, Loko MA, Poizot-Martin I, Valantin MA, Bonnard P, Salmon-Ceron D *et al*: **Role of treatment for depressive symptoms in relieving the impact of fatigue in HIV-HCV co-infected patients: ANRS Co13 Hepavih, France, 2006-2008**. *J Viral Hepat* 2010, **17**(9):650-660.

7. Oberti F, Anty R, Vanbiervliet G, Lacave-Oberti N, Gelsi E, Rosenthal A, Saint Paul M, Rousselet M, Michalak S, Gallois Y *et al*: **Meta-analysis of blood scores of liver fibrosis (Fibrometer, Hepascore, APRI) in alcoholic chronic liver diseases** *Hepatology* 2006, **44**(4Suppl1):467A.

8. Halfon P, Bacq Y, De Muret A, Penaranda G, Bourliere M, Ouzan D, Tran A, Botta D, Renou C, Brechot MC *et al*: **Comparison of test performance profile for blood tests of liver fibrosis in chronic hepatitis C**. *J Hepatol* 2007, **46**(3):395-402.

**Table S1**: **Glossary of fibrosis classifications.**

| **Type** | **Liver specimen** | **Non-invasive test** | **Notes** |
| --- | --- | --- | --- |
| Detailed | Fibrosis stages | Fibrosis classes | A class includes one or several stages (e.g. F_M_) |
| Binary | Significant fibrosis | Significant fibrosis | Two classes: F_M_0/1, F_M_2/3/4.  Other binary classifications: cirrhosis, severe fibrosis (F_M_0/1/2 vs F_M_3/4). |

**Table S2**: **Main characteristics of additional populations.**

| **Population** | **Study name** | **n pts** | **Cause** | **Liver biopsy** | **Blood tests** | **Metavir F prevalence (%)** | | | | |
| --- | --- | --- | --- | --- | --- | --- | --- | --- | --- | --- |
|  |  |  |  |  |  | 0 | 1 | 2 | 3 | 4 |
| #6 | Sniff 14 | 444 | HVC/HIV | x | x | 5.9 | 24.3 | 36.5 | 19.6 | 13.7 |
| #7 | Sniff 29 | 235 | NAFLD | x | x | 43.4 | 28.9 | 8.9 | 8.1 | 10.6 |
| #8 | Sniff 25 | 241 | Alcohol | x | x | 12.6 | 17.9 | 16.8 | 11.6 | 41.1 |

x: test performed

**Table S3**: **Rates of correct classification (%, bold characters) as a function of diagnostic means in populations #6 to #8 with miscellaneous causes.**

|  |  | **Fibrosis class classification**  **(based on F_M_****)** | **Correct classification (%)** | | **p** ^a^ |
| --- | --- | --- | --- | --- | --- |
| **Population** | **#** |  | **Significant fibrosis** **(F_M_≥2)** | **Fibrosis class classification** |  |
| HIV/HCV | 6 | 0/1, 1±1, 1/2, 3±1, 3/4 | **78.7** | **81.6** | 0.542 |
| NAFLD | 7 | 0, 0/1, 1±1, 3±1, 3/4 | **91.1** | **89.8** | 0.549 |
| ALD | 8 | 0, 0/1, 1/2, 2/3, 4 | **88.6** | **77.3** | 0.009 |

NAFLD: non-alcoholic fatty liver disease, ALD: alcoholic liver disease

^a^ by paired McNemar test.

**Figure S1:** **Comparison of patients correctly classified, according to liver biopsy with Metavir fibrosis stages read by expert pathologist (Y axis).**

Comparison with: **1)** local pathologists in population #1 (5 Metavir fibrosis stages, panel **A**); **2)** *fibrosis class classifications* of FibroMeter^2G^ (6 classes, panel **B**), or FibroMeter^3G^ (7 classes, panel **C**) and Fibrotest (8 classes, panel **D**) expressed in Metavir-based fibrosis stages on X axis in population #2.

**Figure S2:** **Comparison of the mean rate of patients correctly classified (Y axis) for fibrosis (F) by *fibrosis class classifications* of FibroMeter^2G^, Fibrotest and Fibroscan in population #3.**

Panels **A** to **C**: comparison as a function of *fibrosis class classifications* of FibroMeter^2G^ (6 classes, panel **A**), Fibrotest (8 classes, panel **B**) and Fibroscan (6 classes, panel **C**) on X axis. Panel **D**: comparison of *fibrosis class classifications* (F_M_, continuous lines) and significant fibrosis (SF, dashed lines) as a function of Metavir F stages by liver biopsy on X axis. FM: FibroMeter^2G^, FT: Fibrotest, FS: Fibroscan.
